# Supplementary material for: A Likelihood Approach for Real-Time Calibration of Stochastic Compartmental Epidemic Models
Source: PLoS Comput Biol. 2017 Jan 17;13(1):e1005257. doi: 10.1371/journal.pcbi.1005257 (PMC5240920; doi:10.1371/journal.pcbi.1005257)
Supplement: S1 File — (TAR.GZ) [file pcbi.1005257.s014.tar.gz › HSPH_Online-SI-Revision/IPoi09/n1-mild/n1-mild_BENCH_table-4.pdf]

1	R0	{1.27677, 7.89375}	{1.82447, 13.8906}	{{1.54207, 9.30931}, {2.12588, 17.7287}}	{42.8972, 76.2797}	inf	17.	80.6071	{13., 151.}	388.866	0.632045	newly	24.	41.5179	{7., 76.}	104.539	0.385127	Reff	1.16697	1.44249	{0.964479, 1.87443}	30.7694	ar	400.	731.393	{579., 844.}	86.2054
2	R0	{1.33231, 10.5259}	{1.88401, 10.7874}	{{1.83592, 9.84456}, {1.98732, 12.1446}}	{41.4091, 7.37484}	inf	39.	51.5273	{14., 90.}	63.0769	0.244051	newly	28.	28.9818	{8., 53.}	47.6623	0.238798	Reff	0.927288	0.54787	{0.408933, 0.656207}	40.9169	ar	467.	765.327	{729., 806.}	63.8816
3	R0	{1.2273, 6.76944}	{1.29471, 2.0993}	{{1.27121, 1.91565}, {1.31246, 2.23442}}	{5.49241, 68.9886}	inf	19.	10.8621	{0., 27.}	63.5209	0.776856	newly	22.	30.2414	{0., 64.}	110.815	0.802984	Reff	1.11071	0.889931	{0.663572, 1.15628}	21.3	ar	314.	362.069	{141., 537.}	37.5577
4	R0	{1.41215, 17.5742}	{1.58319, 11.3101}	{{1.35478, 6.88118}, {1.85253, 15.9062}}	{13.4107, 36.7533}	inf	31.	70.3421	{17., 121.}	145.586	0.485763	newly	15.	38.1842	{10., 58.}	170.351	0.507068	Reff	1.27376	1.16691	{0.821107, 1.45799}	16.2133	ar	465.	625.211	{494., 778.}	44.1879
5	R0	{1.42222, 6.15886}	{1.43666, 4.20963}	{{1.40116, 4.00571}, {1.49267, 4.47101}}	{2.21957, 31.6493}	inf	27.	27.6944	{7., 46.}	50.7202	0.343107	newly	31.	40.8333	{8., 78.}	70.7885	0.381247	Reff	1.21173	0.807505	{0.623258, 1.0583}	34.0373	ar	490.	533.444	{473., 601.}	14.9433
6	R0	{1.24363, 9.95596}	{1.57425, 12.236}	{{1.11461, 5.72868}, {2.12588, 18.9248}}	{30.6094, 47.1596}	inf	12.	11.3478	{1., 25.}	77.8986	0.526166	newly	7.	7.65217	{0., 20.}	93.7888	0.629997	Reff	1.21503	1.60327	{1.13559, 2.14317}	34.7446	ar	411.	457.848	{4., 807.}	77.843
7	R0	{1.36478, 5.96056}	{1.40798, 3.17957}	{{1.37363, 2.81014}, {1.47608, 3.83841}}	{3.25529, 46.6566}	inf	11.	21.1786	{3., 59.}	126.948	0.393544	newly	13.	34.4286	{3., 96.}	190.11	0.489237	Reff	1.2092	0.863454	{0.631921, 1.21429}	30.1159	ar	484.	473.929	{347., 586.}	17.9752
8	R0	{1.36335, 17.6429}	{1.55323, 17.5536}	{{1.42775, 14.2967}, {1.65054, 19.8898}}	{14.0043, 10.3967}	inf	30.	49.7073	{6., 88.}	104.715	0.400375	newly	14.	20.3171	{3., 38.}	89.3728	0.409612	Reff	1.27473	1.26692	{0.983363, 1.52807}	12.3859	ar	484.	586.317	{524., 681.}	31.1177
9	R0	{1.26575, 5.28707}	{1.55714, 9.31416}	{{1.51295, 8.4085}, {1.6568, 10.5289}}	{23.0209, 76.1686}	inf	13.	48.975	{24., 86.}	278.269	0.526756	newly	20.	34.325	{16., 54.}	80.375	0.233298	Reff	1.05943	0.851046	{0.593697, 1.17352}	26.4991	ar	472.	605.625	{494., 700.}	31.1282
10	R0	{1.42942, 9.49905}	{2.03856, 15.6227}	{{1.81743, 12.6353}, {2.26161, 19.3849}}	{42.6146, 64.4659}	inf	45.	128.323	{63., 201.}	186.595	0.418357	newly	34.	53.4194	{24., 81.}	68.5009	0.222639	Reff	1.07635	0.813473	{0.471817, 1.12141}	30.1537	ar	468.	804.726	{742., 867.}	71.95
11	R0	{1.46827, 15.0983}	{1.45932, 15.5518}	{{1.31242, 10.6103}, {1.59433, 19.1171}}	{5.95412, 18.5084}	inf	28.	36.9355	{8., 66.}	69.2396	0.313731	newly	14.	16.9677	{2., 32.}	67.7419	0.33206	Reff	1.36843	1.28436	{1.14473, 1.43819}	9.86378	ar	495.	556.258	{482., 683.}	25.1092
12	R0	{1.25751, 13.279}	{1.21685, 6.8283}	{{1.18976, 5.98888}, {1.25721, 8.10487}}	{4.17947, 48.602}	inf	18.	12.7059	{1., 31.}	60.1307	0.509809	newly	8.	11.8235	{2., 24.}	91.9118	0.403681	Reff	1.17829	0.947712	{0.787713, 1.08891}	19.5867	ar	366.	311.706	{146., 481.}	27.2099
13	R0	{1.3425, 9.16361}	{1.8252, 9.68471}	{{1.71337, 8.80249}, {1.88096, 10.6367}}	{35.9556, 7.71717}	inf	32.	64.9636	{29., 120.}	108.693	0.282245	newly	28.	42.8909	{19., 80.}	70.4545	0.217895	Reff	0.965258	0.689664	{0.447436, 1.04393}	34.7841	ar	442.	739.727	{680., 787.}	67.3591
14	R0	{1.18936, 6.20583}	{1.77729, 9.10085}	{{1.58886, 7.28757}, {1.95639, 11.2959}}	{49.4327, 48.7848}	inf	26.	93.	{32., 154.}	268.524	0.583319	newly	32.	69.449	{35., 99.}	129.528	0.40727	Reff	1.01333	1.21021	{0.772846, 1.61229}	30.6604	ar	382.	707.551	{581., 804.}	89.08
15	R0	{1.27137, 9.78868}	{1.42817, 4.94622}	{{1.38889, 4.24222}, {1.47651, 5.65591}}	{12.3328, 49.47}	inf	27.	29.9143	{10., 57.}	50.582	0.233794	newly	24.	36.9143	{14., 68.}	76.4286	0.250013	Reff	1.10101	0.925736	{0.680957, 1.30387}	23.1845	ar	496.	516.2	{402., 600.}	13.0818
16	R0	{1.23102, 13.6452}	{1.94151, 15.212}	{{1.68954, 11.6019}, {2.2194, 19.3849}}	{57.7157, 20.3807}	inf	33.	105.172	{30., 174.}	228.109	0.486829	newly	16.	50.7931	{18., 73.}	225.862	0.495997	Reff	1.07222	1.37662	{0.986408, 1.76665}	32.7869	ar	445.	774.052	{693., 850.}	73.9442
17	R0	{1.27213, 13.7452}	{1.68201, 12.3032}	{{1.34436, 6.71734}, {1.99753, 17.7287}}	{32.3551, 26.1451}	inf	22.	67.32	{8., 131.}	223.636	0.581047	newly	11.	37.78	{12., 61.}	258.	0.616777	Reff	1.19962	1.3541	{0.965388, 1.72964}	22.142	ar	367.	629.82	{254., 836.}	88.2234
18	R0	{1.25318, 11.2151}	{1.64564, 14.7589}	{{1.53432, 11.4771}, {1.80508, 18.4377}}	{31.3173, 32.1216}	inf	26.	58.8163	{24., 97.}	138.148	0.372531	newly	13.	29.9796	{8., 46.}	143.799	0.382307	Reff	1.10029	1.27144	{0.883413, 1.60359}	23.3159	ar	416.	630.245	{531., 739.}	55.2002
19	R0	{1.46006, 8.11496}	{2.46765, 15.9423}	{{2.0968, 12.1677}, {2.85956, 19.898}}	{69.0102, 96.4562}	inf	42.	121.776	{30., 224.}	202.594	0.440594	newly	40.	60.3582	{17., 109.}	77.0149	0.274629	Reff	1.24251	1.86144	{1.34451, 2.40581}	50.587	ar	496.	879.597	{810., 928.}	77.3381
20	R0	{1.14273, 6.71261}	{1.89948, 15.071}	{{1.6568, 10.8293}, {2.12978, 19.2116}}	{66.2233, 124.518}	inf	15.	80.8246	{10., 147.}	450.994	0.685046	newly	12.	40.2807	{6., 72.}	251.754	0.524321	Reff	1.02046	1.51726	{1.09827, 1.85634}	50.7936	ar	362.	755.93	{629., 863.}	111.971
21	R0	{1.42432, 8.23905}	{1.47788, 3.13061}	{{1.4804, 3.12918}, {1.4804, 3.12918}}	{3.89414, 62.0028}	inf	17.	2.9	{0., 5.}	87.6471	1.69897	newly	14.	4.3	{0., 7.}	95.	1.6227	Reff	1.15512	0.706447	{0.55663, 0.836426}	38.8423	ar	405.	535.6	{411., 611.}	32.2469
22	R0	{1.29386, 17.863}	{1.77804, 14.3118}	{{1.53432, 10.2395}, {2.036, 19.2116}}	{37.465, 22.666}	inf	27.	65.2353	{12., 128.}	160.058	0.408793	newly	8.	34.1961	{9., 61.}	337.255	0.582455	Reff	1.20588	1.48147	{1.11256, 1.83925}	26.5858	ar	488.	720.882	{620., 810.}	47.7218
23	R0	{1.39274, 13.0167}	{1.60356, 8.47813}	{{1.56097, 8.12461}, {1.62191, 8.7843}}	{15.1371, 34.8673}	inf	34.	16.1951	{2., 36.}	65.4232	0.689005	newly	12.	12.0732	{1., 27.}	82.7236	0.577092	Reff	1.12533	0.618016	{0.499548, 0.79362}	45.0815	ar	475.	652.732	{595., 708.}	37.4172
24	R0	{1.32178, 15.8991}	{1.74045, 15.0407}	{{1.53432, 10.7905}, {1.98971, 19.3194}}	{31.6748, 17.5171}	inf	43.	76.8077	{22., 142.}	102.147	0.321043	newly	19.	36.2692	{11., 58.}	111.134	0.344405	Reff	1.16978	1.31643	{0.946126, 1.6541}	22.8818	ar	471.	686.615	{565., 806.}	49.4284
25	R0	{1.37568, 17.8427}	{1.45621, 9.02229}	{{1.36632, 6.88118}, {1.57414, 11.4771}}	{6.12435, 49.4343}	inf	18.	42.2162	{10., 83.}	149.85	0.356618	newly	6.	26.7838	{9., 43.}	349.099	0.597211	Reff	1.20647	0.890034	{0.654631, 1.28771}	28.4536	ar	398.	558.135	{444., 676.}	43.8069
26	R0	{1.20993, 6.4656}	{1.81225, 12.5476}	{{1.61188, 9.30931}, {2.00049, 16.292}}	{49.7812, 94.0664}	inf	13.	88.6809	{19., 151.}	596.236	0.822902	newly	16.	49.7872	{10., 82.}	228.989	0.616458	Reff	1.03933	1.30187	{0.816948, 1.73408}	33.3602	ar	345.	736.447	{642., 824.}	113.463
27	R0	{1.20548, 13.8003}	{1.79683, 18.9268}	{{1.70497, 15.8627}, {1.84468, 19.9256}}	{49.0552, 37.229}	inf	42.	77.6981	{25., 136.}	105.031	0.314771	newly	24.	30.0943	{10., 52.}	61.5566	0.267502	Reff	1.0548	1.38625	{1.0295, 1.67024}	33.5275	ar	350.	724.057	{660., 776.}	106.873
28	R0	{1.3176, 14.6594}	{1.49859, 14.7297}	{{1.19093, 7.7153}, {1.75814, 19.2972}}	{16.9597, 23.1087}	inf	14.	23.0714	{3., 52.}	115.816	0.471302	newly	4.	11.3571	{0., 27.}	231.548	0.618539	Reff	1.27412	1.42036	{1.16495, 1.70252}	17.1842	ar	362.	546.452	{241., 741.}	70.7643
29	R0	{1.28202, 6.23047}	{1.78839, 13.8043}	{{1.58389, 10.5289}, {2.00043, 17.4222}}	{39.4978, 121.562}	inf	36.	91.1	{19., 146.}	168.5	0.425171	newly	35.	46.22	{13., 69.}	56.5143	0.239948	Reff	1.10382	1.18679	{0.790257, 1.67106}	25.6925	ar	391.	714.8	{598., 815.}	82.8133
30	R0	{1.28177, 8.83252}	{1.99859, 11.7285}	{{1.58886, 7.29751}, {2.46976, 16.1789}}	{55.9241, 39.1858}	inf	30.	110.373	{22., 186.}	281.765	0.565584	newly	21.	65.7059	{16., 102.}	227.824	0.521195	Reff	1.12539	1.4592	{0.992823, 2.07497}	36.3446	ar	427.	802.196	{718., 895.}	87.8679
31	R0	{1.35886, 5.97708}	{1.90384, 11.3166}	{{1.67405, 7.29751}, {2.1255, 14.3165}}	{40.1056, 89.3325}	inf	23.	101.431	{43., 180.}	345.502	0.599149	newly	44.	65.6379	{29., 103.}	65.1646	0.234158	Reff	1.14008	1.24465	{0.787414, 1.87276}	29.3254	ar	491.	752.655	{629., 844.}	53.2903
32	R0	{1.27601, 17.9804}	{1.67025, 16.5381}	{{1.46004, 12.7726}, {1.8189, 19.688}}	{30.8966, 14.1137}	inf	25.	57.6957	{4., 123.}	162.957	0.559727	newly	8.	25.8913	{1., 48.}	252.989	0.667734	Reff	1.18924	1.30946	{0.957211, 1.6874}	19.7889	ar	445.	621.543	{513., 773.}	55.5398
33	R0	{1.3368, 11.3235}	{1.77954, 13.3978}	{{1.52414, 9.08517}, {2.00049, 17.4222}}	{33.1191, 27.4483}	inf	40.	71.3617	{31., 134.}	90.9574	0.26383	newly	21.	38.6596	{18., 63.}	95.0355	0.281167	Reff	1.1697	1.43102	{1.00235, 1.79229}	28.014	ar	417.	722.787	{610., 806.}	73.3303
34	R0	{1.21021, 11.3635}	{1.36434, 5.2159}	{{1.31748, 4.25564}, {1.40191, 6.71734}}	{12.736, 54.0996}	inf	20.	10.8125	{0., 29.}	63.125	0.846702	newly	14.	13.2813	{0., 35.}	81.4732	0.79141	Reff	1.06862	0.829689	{0.679538, 1.06966}	24.2181	ar	327.	442.844	{307., 543.}	39.8987
35	R0	{1.53382, 10.8866}	{2.00781, 16.1719}	{{1.73739, 12.272}, {2.26107, 19.5128}}	{30.9023, 49.3076}	inf	30.	78.7167	{16., 138.}	181.389	0.46497	newly	22.	37.85	{7., 67.}	97.803	0.359116	Reff	1.3789	1.64534	{1.25231, 1.95921}	23.5036	ar	476.	763.867	{676., 854.}	66.8557
36	R0	{1.20423, 6.83844}	{2.21843, 15.0885}	{{1.85376, 10.0119}, {2.53519, 19.391}}	{84.2199, 120.643}	inf	44.	83.0167	{21., 143.}	109.28	0.321103	newly	49.	45.7833	{14., 82.}	46.3605	0.280656	Reff	1.05852	1.84166	{1.3408, 2.27096}	73.9849	ar	416.	834.867	{727., 898.}	100.689
37	R0	{1.43629, 10.1548}	{1.39116, 6.50181}	{{1.3618, 4.98002}, {1.42446, 7.81552}}	{3.27905, 36.0983}	inf	15.	20.4643	{1., 45.}	94.0476	0.47198	newly	13.	19.	{1., 39.}	104.396	0.499265	Reff	1.27255	0.862394	{0.601122, 1.27384}	32.6964	ar	367.	469.357	{315., 582.}	37.7579
38	R0	{1.37504, 8.93428}	{2.16713, 12.7099}	{{1.77826, 9.06807}, {2.64334, 17.2462}}	{57.6047, 45.4432}	inf	32.	138.344	{43., 221.}	333.984	0.579364	newly	24.	77.6875	{35., 113.}	226.042	0.481856	Reff	1.17703	1.41105	{0.83307, 2.236}	34.4463	ar	483.	831.016	{680., 925.}	72.0529
39	R0	{1.51637, 9.36742}	{1.63682, 6.96536}	{{1.54465, 5.30296}, {1.68297, 7.5059}}	{8.4167, 25.7251}	inf	43.	49.5	{19., 88.}	48.2216	0.206286	newly	31.	44.5294	{16., 76.}	70.019	0.25521	Reff	1.204	0.819886	{0.548648, 1.22107}	34.6989	ar	441.	649.441	{556., 721.}	47.2656
40	R0	{1.23658, 9.82869}	{1.37338, 3.42906}	{{1.31023, 3.04996}, {1.42949, 4.24222}}	{11.063, 65.1118}	inf	29.	19.24	{3., 40.}	51.0345	0.4639	newly	15.	34.36	{3., 73.}	161.6	0.499893	Reff	1.09561	0.8177	{0.62304, 1.091}	27.2055	ar	414.	487.04	{344., 608.}	27.6329
41	R0	{1.24247, 5.4242}	{2.16735, 12.0014}	{{1.55618, 5.4396}, {2.74988, 17.2462}}	{74.4385, 124.052}	inf	23.	58.25	{13., 110.}	173.841	0.415062	newly	36.	42.6833	{6., 75.}	61.0648	0.289852	Reff	1.1381	1.93816	{1.412, 2.5187}	71.8391	ar	345.	814.25	{642., 927.}	136.014
42	R0	{1.28208, 4.40223}	{1.38684, 5.44433}	{{1.22544, 3.26445}, {1.6082, 8.77064}}	{9.7951, 39.1044}	inf	20.	31.5455	{6., 63.}	102.273	0.421787	newly	30.	37.6364	{5., 67.}	69.4949	0.37835	Reff	1.1949	1.17009	{0.846228, 1.51418}	20.1483	ar	339.	477.394	{131., 693.}	63.3861
43	R0	{1.56773, 6.50474}	{1.92631, 6.25933}	{{1.61691, 4.30394}, {2.42049, 9.16315}}	{23.4551, 28.5603}	inf	33.	98.5789	{39., 197.}	204.997	0.428183	newly	44.	113.	{64., 173.}	160.965	0.393164	Reff	1.34982	1.3605	{0.768879, 2.03913}	26.5777	ar	498.	756.509	{602., 892.}	51.9094
44	R0	{1.10489, 6.01941}	{1.85203, 14.3678}	{{1.68954, 11.6019}, {2.00043, 17.4222}}	{67.6211, 138.691}	inf	12.	107.385	{43., 148.}	798.718	0.946397	newly	14.	53.3846	{25., 78.}	288.462	0.598802	Reff	0.942471	1.14593	{0.85326, 1.55395}	27.1641	ar	346.	728.942	{660., 818.}	113.956
45	R0	{1.25888, 14.1926}	{1.74288, 12.811}	{{1.64295, 10.5289}, {1.8951, 15.9062}}	{38.4465, 13.9509}	inf	50.	82.3529	{39., 119.}	73.2549	0.23315	newly	23.	42.7647	{26., 62.}	89.3436	0.261354	Reff	0.981926	0.912432	{0.631615, 1.29957}	24.4037	ar	471.	697.706	{610., 774.}	48.1329
46	R0	{1.45292, 16.616}	{1.99531, 15.5423}	{{1.6568, 10.7905}, {2.27287, 19.3211}}	{37.4445, 15.1808}	inf	34.	72.1231	{6., 146.}	146.154	0.506387	newly	17.	35.1385	{2., 67.}	140.724	0.513595	Reff	1.34105	1.7118	{1.37072, 2.06888}	29.9913	ar	494.	767.246	{694., 873.}	64.17
47	R0	{1.22528, 16.6033}	{1.55496, 13.6525}	{{1.32705, 8.42022}, {1.80508, 19.2972}}	{26.9068, 24.5851}	inf	14.	32.8	{8., 66.}	155.102	0.37757	newly	6.	17.3714	{4., 38.}	210.476	0.47239	Reff	1.17014	1.50852	{1.16927, 1.74079}	30.2583	ar	368.	638.314	{524., 746.}	79.014
48	R0	{1.1669, 6.92715}	{1.54737, 9.54658}	{{1.51295, 8.4085}, {1.58389, 10.8293}}	{32.6051, 37.8139}	inf	17.	50.6154	{23., 91.}	198.944	0.433084	newly	17.	34.8974	{17., 59.}	108.597	0.285813	Reff	0.977862	0.865947	{0.582486, 1.27456}	22.9952	ar	416.	619.923	{533., 690.}	49.02
49	R0	{1.32749, 16.0887}	{1.44676, 11.9494}	{{1.19306, 6.16288}, {1.75588, 18.8228}}	{12.554, 30.8375}	inf	24.	39.7059	{8., 77.}	101.225	0.382982	newly	8.	22.1765	{6., 42.}	194.853	0.496253	Reff	1.24917	1.26943	{0.915531, 1.66441}	15.4891	ar	353.	559.971	{365., 738.}	66.4306
50	R0	{1.29636, 15.2609}	{1.91931, 15.8679}	{{1.73739, 12.378}, {2.11476, 19.2116}}	{48.0541, 12.965}	inf	50.	112.961	{56., 149.}	138.471	0.399181	newly	20.	49.8235	{24., 69.}	160.294	0.426139	Reff	1.03838	1.22411	{0.818228, 1.66405}	28.0329	ar	470.	752.216	{676., 826.}	63.6587
